# A combination of circulating microRNA-375-3p and chemokines CCL11, CXCL12, and G-CSF differentiate Crohn’s disease and intestinal tuberculosis

**DOI:** 10.1038/s41598-021-02383-z

**Published:** 2021-12-02

**Authors:** Susree Roy, Suchandrima Ghosh, Mallica Banerjee, Sayantan Laha, Dipanjan Bhattacharjee, Rajib Sarkar, Sujay Ray, Arko Banerjee, Ranajoy Ghosh, Aniket Halder, Alakendu Ghosh, Raghunath Chatterjee, Simanti Datta, Gopal Krishna Dhali, Soma Banerjee

**Affiliations:** 1 Center for Liver Research, School of Digestive and Liver Diseases, Institute of Post Graduate Medical Education and Research, 244, Acharya Jagadish Chandra Bose Road, Kolkata, West Bengal 700020 India; 2 Department of Human Genetics, Indian Statistical Institute, Kolkata, India; 3 Department of Rheumatology, Institute of Post Graduate Medical Education and Research, Kolkata, India; 4 Department of Gastroenterology, School of Digestive and Liver Diseases, Institute of Post Graduate Medical Education and Research, Kolkata, India; 5 R.G. Kar Medical College and Hospital, Kolkata, India; 6 Department of Gastro-Pathology, School of Digestive and Liver Diseases, Institute of Post Graduate Medical Education and Research, Kolkata, India

**Keywords:** Gastrointestinal diseases, Diagnostic markers

## Abstract

Differentiation of Crohn’s disease (CD) from intestinal tuberculosis (ITB) is a big challenge to gastroenterologists because of their indistinguishable features and insensitive diagnostic tools. A non-invasive biomarker is urgently required to distinguish ITB/CD patients particularly in India, a TB endemic region, where CD frequency is increasing rapidly due to urbanization. Among the three differentially expressed miRNAs obtained from small RNA transcriptomic profiling of ileocaecal/terminal ileal tissue of ITB/CD patients (n = 3), only two down-regulated miRNAs, miR-31-5p, and miR-215-5p showed comparable data in qRT-PCR. Out of which, only miR-215-5p was detectable in the patient’s plasma, but there was no significant difference in expression between ITB/CD. On the other hand, miR-375-3p, the pulmonary TB specific marker was found in higher amount in the plasma of ITB patients than CD while reverse expression was observed in the ileocaecal/terminal ileal tissues of the same patients. Next, using Bioplex pro-human cytokine 48-plex screening panel, only three chemokines, Eotaxin-1/CCL11, SDF-1α/CXCL12, and G-CSF have noted significantly different levels in the serum of ITB/CD patients. ROC analysis has revealed that compared to a single molecule, a combination of miR-375-3p + Eotaxin-1/CCL11 + SDF-1α /CXCL12 + G-CSF showed a better AUC of 0.83, 95% CI (0.69–0.96) with 100% specificity and positive predictive value while sensitivity, negative predictive value, and accuracy were 56%, 69%, and 78% respectively in distinguishing ITB from CD. This study suggests that a combination of plasma markers shows better potential in differentiating ITB from CD than a single marker and this panel of markers may be used for clinical management of ITB/CD patients.

## Introduction

Intestinal tuberculosis (ITB) and Crohn’s disease (CD) are two very indistinguishable chronic granulomatous disorders of the gastrointestinal (GI) tract because of their similarities in clinical, radiological, and histological features^1–7^. CD is the outcome of an aberrant immune response to the environmental factors and altered gut microbiota in the genetically predisposed individual while ITB is an infectious condition due to *Mycobacterium tuberculosis*. Despite distinct disease prognosis of ITB and CD, rising incidences of both CD with the global urbanization and ITB due to HIV pandemic, the diagnostic dilemma between these two diseases turns out to be tremendous challenging to the clinicians^8,9^. Misdiagnosis of the disease often delays the initiation of the proper treatment and unnecessary exposure of the patients to either anti-tubercular therapy (ATT) or steroids, which enhances morbidity and mortality^10^. IBD burden is rising sharply in India, which is a TB endemic region. A single-center Indian study with 11,746 autopsies depicted that 3.7% of patients were having abdominal tuberculosis, which included mainly patients with ileocaecal region while HIV sero-prevalence amongst abdominal tuberculosis patients was 16.6%^10–12^. Hence, the gastroenterologists practicing in this region urgently require a biomarker to distinguish these two groups of patients.

Several studies have identified clinical, serological, immunological, and histological parameters to distinguish these two diseases^2^, but none of them showed potential to be used as marker in differentiating these two diseases. A meta-analysis study with pathological features from biopsy samples revealed that confluent, large, multiple granulomas; granuloma with surrounding cuffing lymphocytes, and caseating necrosis are common in ITB and the diagnostic specificity of these features have been found high (99%, 95%, and 100% respectively) but sensitivity was very poor (38%, 41%, and 21% respectively) although the area under curve (AUC) was high (0.94, 0.90 and 0.99 respectively) in each case^13^. Serological tests with ASCA and pANCA are very commonly used to differentiate CD and ulcerative colitis (UC) in the USA^14^ and ASCA has a better specificity for CD than ITB but with low sensitivity^15,16^. Similarly, neither PCR with isolated DNA nor in situ PCR with TB-specific primer could show high sensitivity in differentiating ITB/CD^17^. In 2018, Tiwari et al*.* first tested the non-invasive biomolecule for similar goal. They showed CD4^+^CD25^+^CD127^-^FOXP3^+^T reg cells in the peripheral blood have high diagnostic potential (75% sensitivity and 90.6% specificity) in separating CD and ITB^4^. Exploring the serum proteomic profiling of 30 CD and 21 ITB samples, Zhang et al. in 2016 has identified two proteins, [Appetite peptide and Lysyl Oxidase Like 2 (LOXL-2)] which are deregulated in ITB/CD and showed high prediction potential with 76% sensitivity and 80% specificity^18^, but none of them were validated due to technical limitations.

In the last two decades, microRNAs (miRNAs) have been shown significant contribution as a disease diagnostic marker because of their high stability, easy availability in biofluids and, representing the disease condition. Blood miRNAs have been established to distinguish two common inflammatory bowel diseases CD and UC e.g. miR-19a, miR-21, miR-31, miR-101, miR-146a, miR-375^19^ and miR-598, miR-642^20^. MicroRNAs have been also studied in pulmonary Tuberculosis (pTB). Alipoor et al*.* in 2018 has reported exosomal miRNAs, miR-484, miR-425 and miR-96 as a marker for pTB^21^, while Lingna, Lyu et al. showed miR-140-3p, miR-3184-5p, and miR-423-3p are responsible for the progression of TB^22^. Fu et al. had established miR-29a-3p as a serum and sputum marker for pTB while miR-375 showed 22 fold higher expression in the serum of pTB patients^23^. Our small RNA profiling with ileocaecal/ileal tissue of ITB and CD patients followed by validation with qRT-PCR revealed that only two microRNAs (miR-215-5p, and miR-31-5p) were significantly downregulated in the ileocaecal/ileal tissue of ITB patients compared to CD. But none of the miRNAs were found significantly altered in the plasma of the same patients. Nevertheless, between two pTB markers (miRNA-375-3p and miR-29a), miR375-3p was noted significantly higher in the plasma of ITB patients, while it was lower in the tissue compared to CD patients. Next, serum cytokine profiling disclosed that among 48 cytokines/chemokines, the level of Stromal cell-derived factor-1α (SDF-1α/CXCL12), Eosinophil chemo-attractant (Eotaxin-1/CCL11), and Granulocyte colony stimulating factor (G-CSF) could be used to differentiate ITB/CD. The ROC analysis showed that the combination of three cytokines and miR-375-3p depicted the highest AUC of 0.83, 90% CI (0.69–0.96) with 100% specificity and positive predictive value (PPV) but 56% sensitivity, 69% negative predictive value (NPV) and with 78% accuracy. Therefore, considering the heterogeneity and complexity of both the diseases, a combination of multiple markers might have better predictive potential than the single one.

## Results

### Clinical, demographic, and histological data of human subjects

The clinical, biochemical, histological and molecular data of the human subjects included in the study are presented in Table 1, and Supplementary Table 1. Fifty-two treatment naïve patients, who fulfilled the inclusion criteria for ITB/CD, were enrolled in the study. In addition, three hemorrhoids and fifteen UC patients were included as control. The age and sex of the included patients in the ITB and CD groups were similar. Gola formation was seen more in ITB than CD (80% vs. 60%). ITB was diagnosed mostly in ileocaecal region (80%) while CD was present in terminal Ileal (40%), ileocaecal (36%), and colonic (4%) region. The CRP level was not significantly different between the two groups. None of the patients had extra-intestinal manifestation. All the ITB patients were positive in multiplex PCR with IS6110 and MBP64 primers while CD patients were negative.

**Table 1:** Demographic and clinical parameters of the included subjects.

Variable	ITB (n = 27)	CD (n = 25)	P-value
**Baseline characteristics**			
Age (yr) (mean ± sd)	32.53 ± 12.02	32.7 ± 12.5	0.97
Sex (F/M)	12/15	10/15	
Duration of disease (month)	10 (2–78)	8 (2–180)	0.5
Fever (Y/N)	20/7	19/6	0.96
Pain abdomen (Y/N)	23/4	21/4	0.99
Weight loss (Y/N)	24/3	20/5	0.97
Gola formation (Y/N)	21/6	15/10	0.17
**Clinical characteristics**			
Location (%)			
Terminal ileal	6 (22)	10 (40)	0.57
Ileocecal	21 (78)	9 (36)	0.02
Colonic	0 (00)	6 (24)	0.77
Hemoglobin (gm/dl) (mean ± sd)	10.68 ± 1.57	9.61 ± 3.4	0.81
ESR (mm) (mean ± sd)	47 ± 23.92	52.1 ± 21.8	0.7
CRP (mg/dl) [median (range)]	1.1 (0.7–43.8)	1.74 (0.3–4.7)	0.60
**Histological status**			
Granulomatous iletis (%)	9 (33)	6 (24)	0.97
Chronic active iletis (%)	0 (00)	7 (28)	0.03
Non-specific inflammation (%)	17 (63)	12 (48)	0.74
TB PCR positive (%)	27(100)	0 (00)	6.30E−11

### Small-RNA transcriptome profiling in colonic tissue of CD and ITB patients

**Figure 1:**
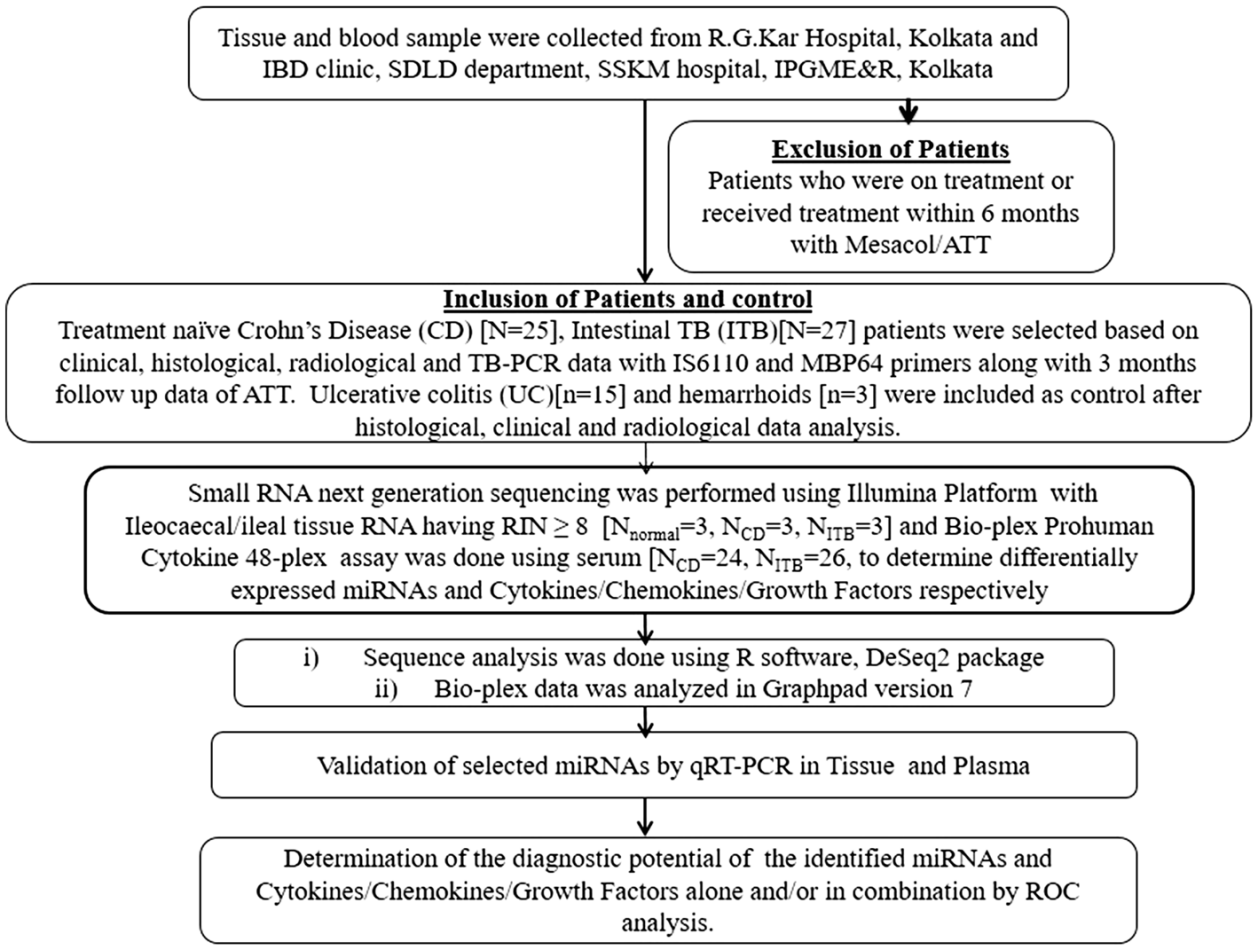
Schematic diagram of experimental flow.

**Figure 2:**
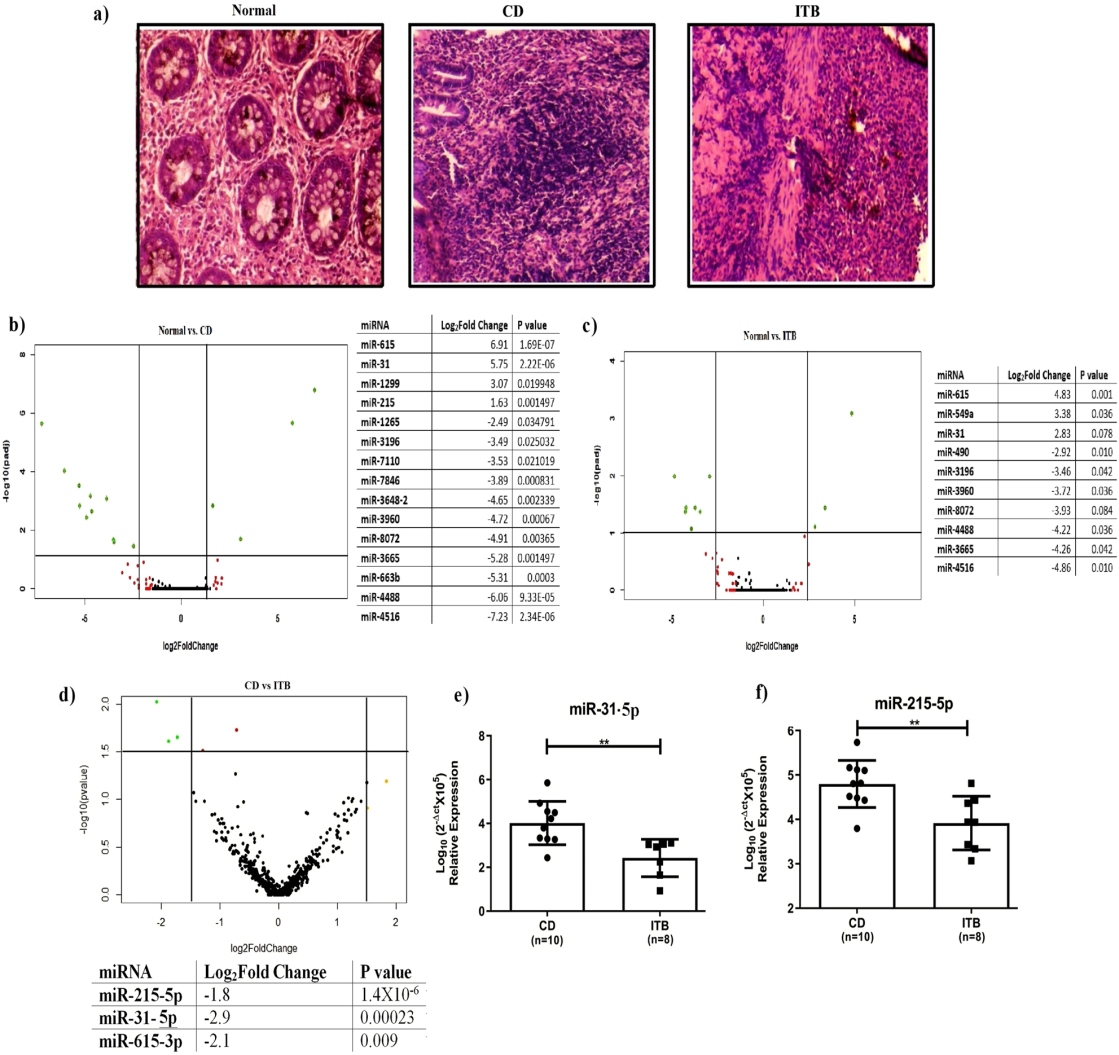
(**a**) Hematoxylin & Eosin staining of ileocaecal/terminal ileal tissue of CD and ITB patients; (**b**–**d**) Volcano plot analysis with NGS data between Control vs. CD, Control vs. ITB and CD vs. ITB. miRNAs showed significant changes in expression are presented in the adjascent tables. Expression of the miRNAs were validated by qRT-PCR (**e**) miR-31-5p and (**f**) miR-215-5p respectively. p-values ≤ 0.01 is presented as **.

The workflow of the study is presented in Fig. 1. The tissue was stained with Hematoxylin and Eosin (Fig. 2a) and two pathologists have scored the disease severity independently. The differentially expressed miRNAs were identified using Illumina high-throughput small RNA sequencing of the ileocaecal/ileal biopsy tissues collected from hemorrhoids (n = 3) as control, ITB (n = 3) and CD (n = 3) patients. After filtering the low quality and polyclonal reads about 85%, and 87% of the data were obtained from CD, and ITB respectively and 98% of each sequence was mapped to the human genome (hg19) (Supplementary Table 2).

The Volcano plots have revealed that fifteen and ten miRNAs showed significant alterations between the control vs. CD group and control vs. ITB group respectively but only three miRNAs showed distinct expression patterns between CD and ITB (Fig. 2b–d). These three miRNAs (miR-215-5p, miR-31-5p, and miR-615-3p) were noted significantly downregulated (p < 0.05) in patients with ITB compared to CD (Fig. 2d). While the expression of the three miRNAs were validated in a larger number of biopsy tissues by qRT-PCR, only two miRNAs (miR-31-5p and miR-215-5p) showed comparable data (Fig. 2e,f).

### Differentially expressed circulating miRNAs in distinguishing ITB and CD

**Figure 3:**
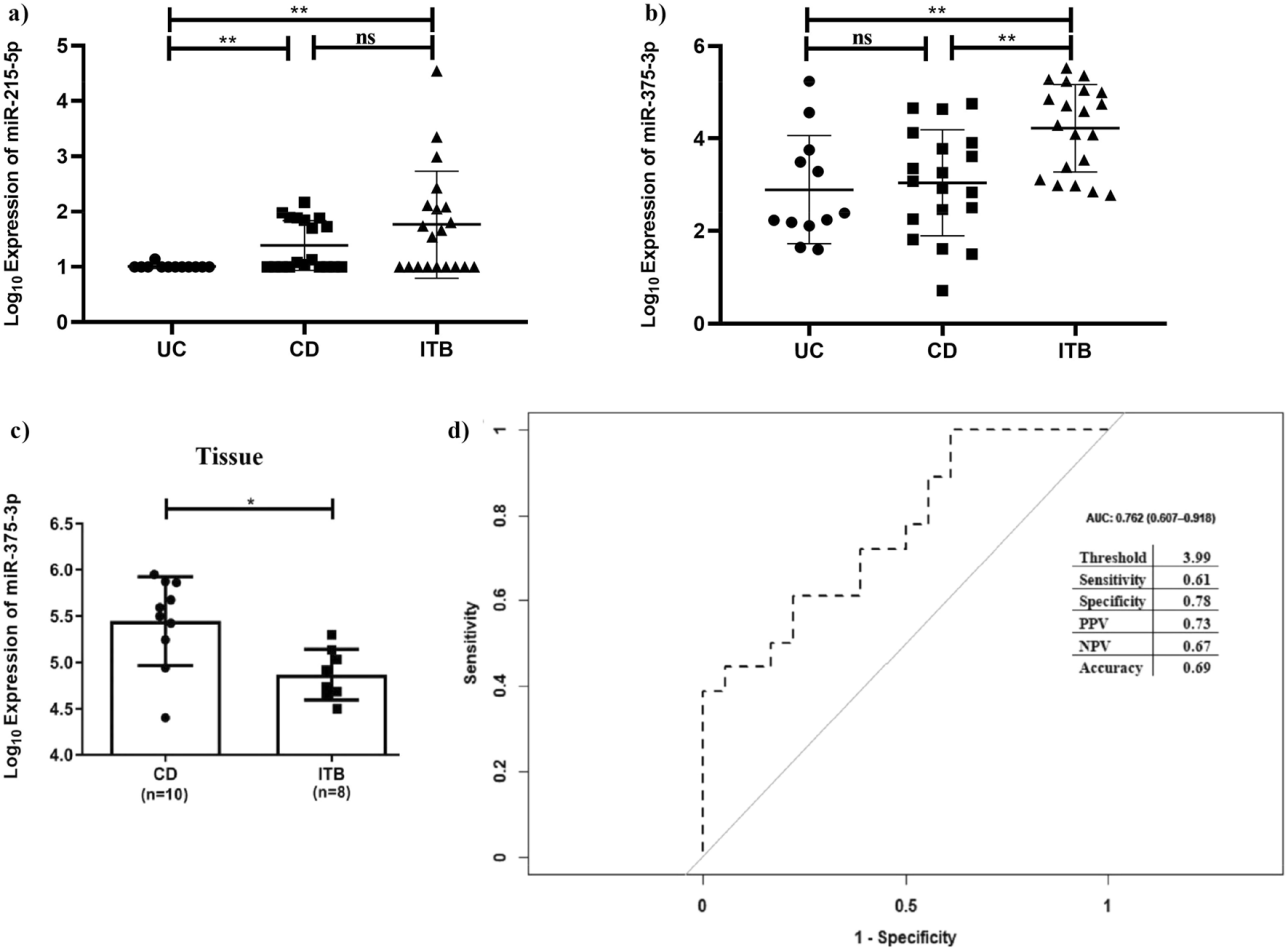
qRT-PCR validation of (**a**) miR-215-5p and (**b**) miR-375-3p in plasma of UC, CD, and ITB patients, and (**c**) miR-375-3p in ileocaecal/ileal tissues of CD and ITB patients and (d) Receiver operating curve analysis of miR-375-3p as marker differentiating CD and ITB and adjacent table includes threshold [Log_10_(2^-Δct^x10^5^)], sensitivity, specificity, positive and negative predictive values. * and ** denotes p-value ≤ 0.05 and 0.01 and ns is not significant.

Now to advance our knowledge in utilizing the two tissue-specific downregulated miRNAs as non-invasive markers to distinguish ITB and CD, the expression pattern of each miRNA was determined in the plasma of the patients by qRT-PCR. To our surprise, miRNA-31-5p was undetectable in the plasma while the expression of miR-215-5p was similar between ITB and CD, albeit it was higher in CD compared to UC (Fig. 3a). Hence, these two miRNAs may be considered as tissue-specific markers for ITB/CD, although validation in large sample cohort is required.

Next, we had validated two pulmonary TB markers, miR-375-3p, and miR-29a in the plasma of the patients and only miR-375-3p was observed significantly higher in the plasma of the ITB patient compared to CD, while miR-29a was undetected in 100% of the samples. There was no difference in expression of miR-375-3p between UC and CD (Fig. 3b). The expression of miR-375-3p was further verified in the tissue samples of both CD and ITB and an opposite expression pattern was observed (Fig. 3c).

### Evaluation of the diagnostic potential of miRNAs as plasma biomarker in separating ITB and CD

Receiver operating curve (ROC) analysis was performed to determine the diagnostic potential of the miRNAs. R script was generated to determine the Area under the curve (AUC) of miR-375-3p and it was observed 0.76, 95% CI of (0.61–0.92), the sensitivity of 61%, and specificity of 78%. The positive and negative predictive values for the detection were 73% and 67% respectively with 69% accuracy (Fig. 3d).

### Deregulated cytokines in differentiating ITB and CD

**Figure 4:**
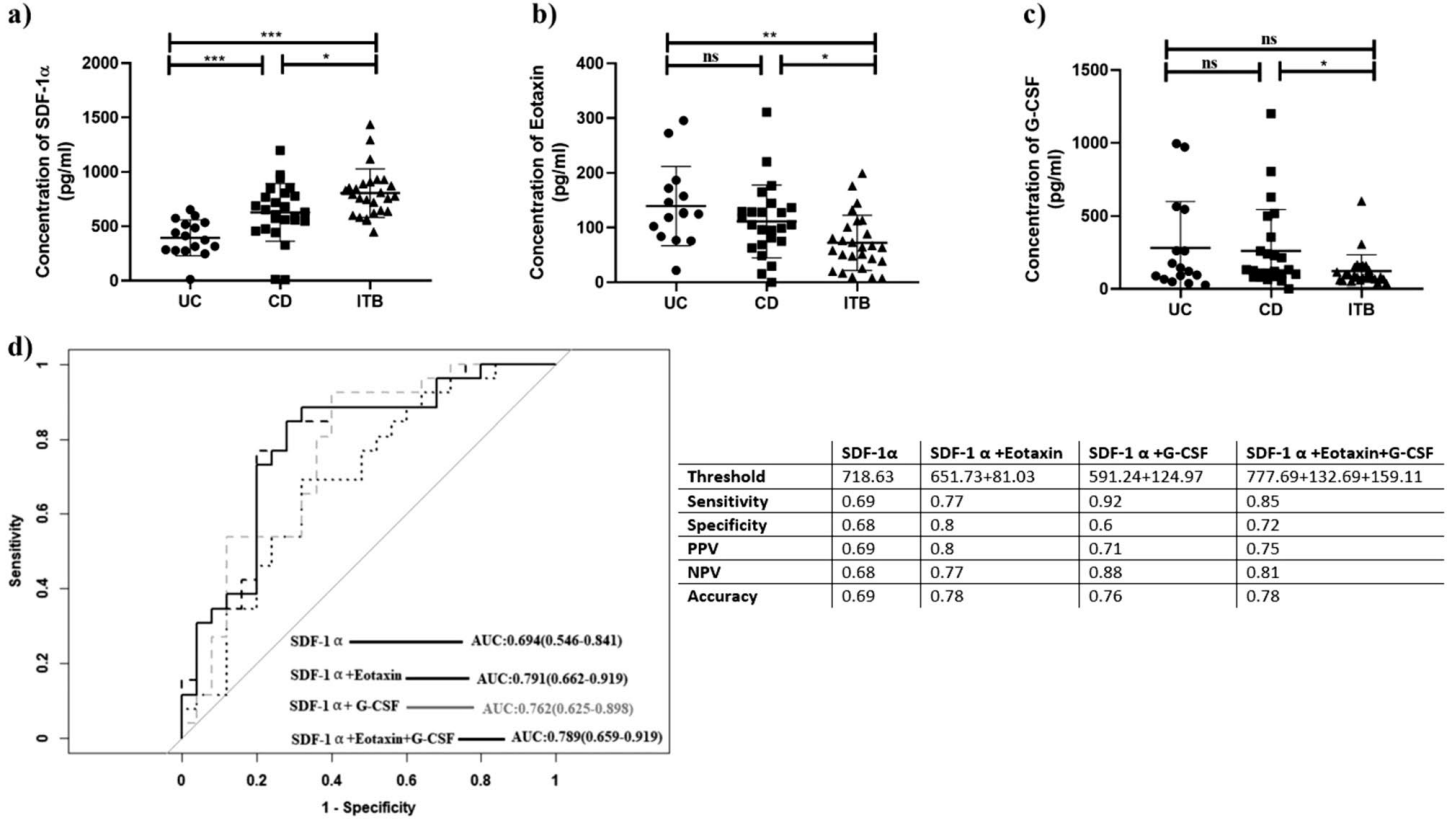
(**a**–**c**) ELISA data of SDF-1α/ CXCL12, Eotaxin-1/CCL11, and G-CSF in serum of UC, CD, and ITB patient. (**d**) ROC analysis of each chemokine and their combinations. Table shows threshold (pg/ml), sensitivity, specificity, positive and negative predictive values. p-values ≤ 0.05 is denoted as *.

To improve the diagnostic potential of the plasma miRNAs, deregulated cytokines and chemokines were also quantified in the serum of ITB and CD patients using Bioplex pro-human cytokine array, which includes 48 chemokines and cytokine cell signaling molecules. Among these 48 signaling molecules, the level of only three chemokines SDF-1α/CXCL12, Eotaxin-1/CCL11, and G-CSF showed significant differences between ITB and CD (Fig. 4a–c). Eotaxin-1/CCL11 and G-CSF were found significantly higher in the CD patients tested in comparison to the ITB patients while SDF-1α /CXCL12 level was higher in ITB than CD. On the other hand, Eotaxin-1/CCL11, and G-CSF could not differentiate UC and CD but the level of SDF-1α /CXCL12 was significantly higher in CD compared to UC (Fig. 4a–c).

Next, ROC analysis was performed to determine the diagnostic potential of the above chemokines in distinguishing ITB and CD patients. In comparison to the AUC of single chemokine, a combination of SDF-1α/CXCL12 + Eotaxin-1/CCL11 showed AUC of 0.79, 90% CI (0.66–0.92) while the addition of G-CSF did not show any further improvement in the AUC value (Fig. 4d). The sensitivity, specificity of this combination was 77%, and 80% respectively while PPV, NPV and accuracy were 80%, 77% and 78% respectively. Again SDF-1α/CXCL12 + G-CSF had AUC of 0.76, 90% CI (0.63–0.90) with 92% sensitivity, 60% specificity, 71% PPV, 88% NPV, and 76% accuracy (Fig. 4d). The combination of three chemokines showed 85% sensitivity and 72% specificity, 75% PPV, 81% NPV and 78% accuracy in differentiating ITB and CD. SDF-1α/CXCL12 could also differentiate CD from UC with AUC of 0.81, 90% CI of (0.68–0.95), sensitivity, specificity of 76%, 81% and NPV, PPV of 86%, 68% respectively (Supplementary Fig. 1).

### Diagnostic potential for the combination of miRNA-375-3p and chemokines

**Figure 5:**
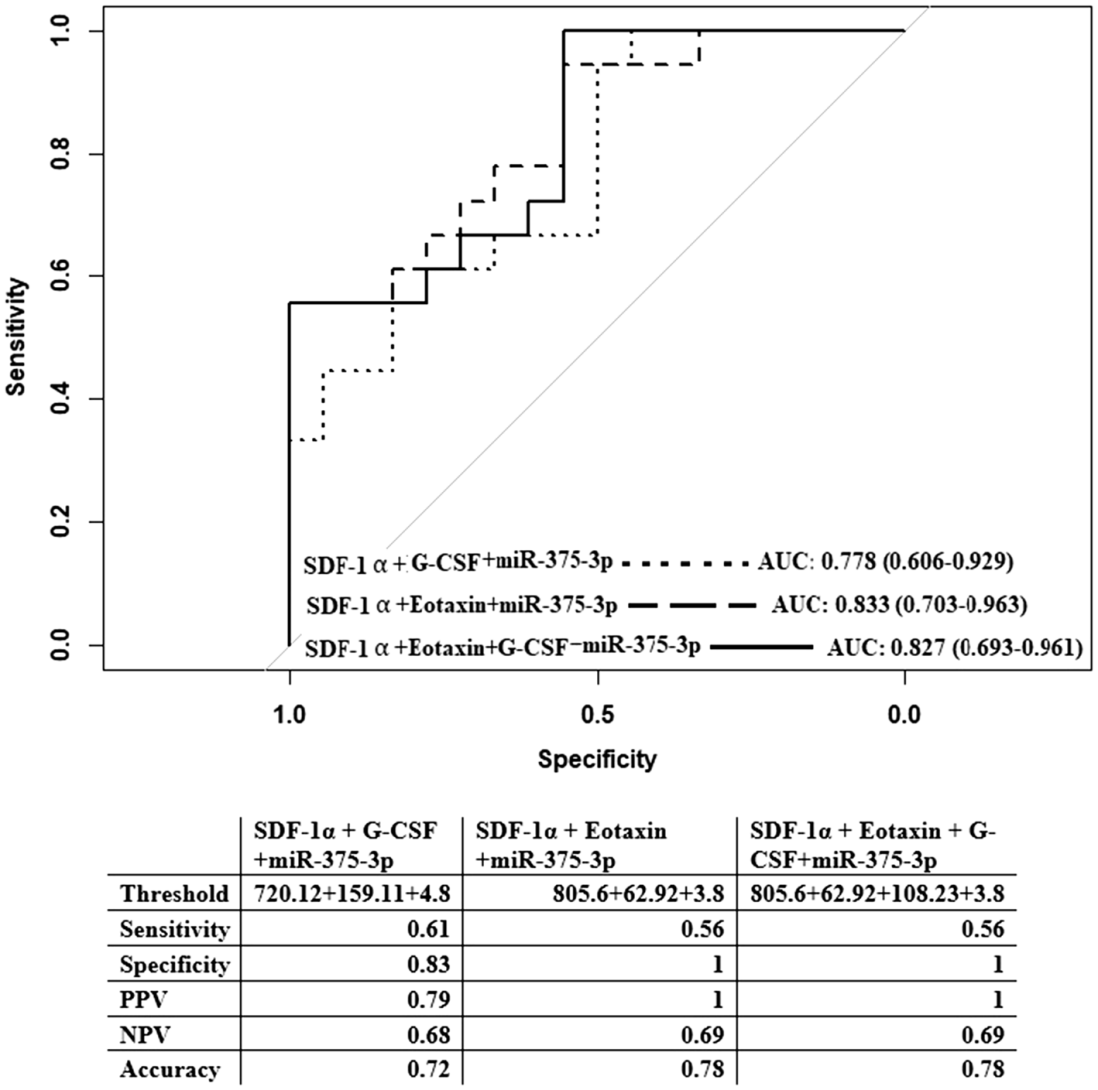
ROC analysis with different combination of all the four markers miR-375-3p + SDF-1α/CXCL12 + G-CSF; miR-375-3p + SDF-1α/CXCL12 + Eotaxin-1/CCL11; and miR-375-3p + Eotaxin-1/CCL11 + SDF-1α/CXCL12 + G-CSF. Threshold (pg/ml for chemokines and [Log_10_(2^-Δct^x10^5^)] for miRNA, sensitivity, specificity, positive and negative predictive values are included in the adjacent table for each comparison.

The diagnostic potentials of SDF-1α/CXCL12 + G-CSF or SDF-1α/CXCL12 + Eotaxin-1/CCL11, and SDF-1α/CXCL12 + Eotaxin-1/CCL11 + G-CSF are better than miR-375-3p alone. ROC analysis was employed for combination of SDF-1α/CXCL12+G-CSF+miR-375-3p, and observed AUC was 0.78, 95% CI (0.61–0.93) with 61% sensitivity, 83% specificity, 79% PPV, 68% NPV, and 72% accuracy. While the combination of either SDF-1α/CXCL12+Eotaxin-1/CCL11+miR-375-3p or SDF-1α/CXCL12+Eotaxin-1/CCL11+G-CSF+miR-375-3p similar AUC of 0.83, Sensitivity of 56%, sensitivity, 100% specificity, PPV of 100%, NPV of 69%, and accuracy of 78% indicating addition of miR-375-3p to the three chemokines improve the overall diagnostic potential in separating ITB and CD (Fig. 5).

## Discussion

The rising incidences of ITB and CD due to misdiagnosis of these two diseases and subsequent treatment of both the groups with the ATT drugs, augments the degree of disease complexity in CD patients. Several clinical studies include radiological investigations, histological appearance in colonoscopic biopsy tissue^1,2^ and immunological parameters^18^ in order to separate these two diseases. But to our knowledge the sensitivity and specificity of each method is not sufficient to be used in diagnosis except CD4^+^CD25^+^CD127^-^FOXP3 + T reg cells^4^ and Appetite peptide, LOXL-2 serum proteins as a non-invasive biomarker^18^, albeit none of them have been validated in large cohorts. Thus, this study further strives to identify a non-invasive biomarker that could separate ITB and CD with high diagnostic potential using two independent molecular approaches. It is now very clear from a myriad of molecular studies that expression of non-coding RNAs such as miRNAs alter swiftly with slight changes in physiological conditions, hence miRNAs are being used widely as molecular diagnostic markers for many diseases including IBD^24,25^ and lung TB^26^. Along with this, cellular cytokines and chemokines milieu also alter with the physiological condition. Hence, a combination of secretary miRNAs and chemokines in blood have been considered in this study to differentiate between these two diseases more efficiently than the previous biomarkers.

This is the first comparative study where ileal/ileocecal tissue-specific miRNAs were evaluated in the blood and also the serum cytokines/chemokines levels in ITB and CD patients and UC was considered as control. The NGS data revealed that only three miRNAs (miR-215-5p, miR-615-3p and miR-31-5p) were differentially regulated with ≥ 1.3-fold alterations in expression between the two diseases. This low number of differentially expressed miRNAs reflects the indistinguishable nature of these two diseases. Unfortunately, none of the downregulated miRNAs were observed in the plasma. We might have missed a few of the deregulated miRNAs as the number of samples used in NGS was very low. Thus, two pulmonary TB markers miR-375-3p, and miR-29a were validated in the plasma and tissue samples and only miR-375-3p was found to be enriched in the plasma of the ITB patients compared to CD. Subsequently, the expression of miR-375-3p was noted less in the ITB tissue than in CD. Schaefer et al. in 2015 had shown that miR-375-3p was significantly reduced in colon biopsy of CD and UC patients than normal while it was enriched in the plasma of both CD and UC relative to normal^19^. But there was no significant difference in expression of miR-375-3p between CD and UC after BonFerroni correction. On the otherhand, the expression of miR-215-5p has been depicted as a plasma prognostic biomarker for CD in several studies. But miR-215-5p was not detected in 100% of the samples used in the study. ROC analysis revealed that miR-375-3p showed only 61% sensitivity and 78% specificity in distinguishing ITB from CD.

Immune markers are also considered as potential biomarkers for different diseases^27,28^. Both CD and ITB are associated with the accumulation of mucosal lymphomononuclear cells, macrophages, etc. and M1 macrophage population remains higher in CD patients than in ITB^28^. Thus, we intended to identify deregulated chemokines that functions as chemo-attractants and cytokines whose main action is to recruit immune cells to the inflammatory sites. Screening of deregulated chemokines in CD and ITB was investigated using a 48 plex Cytokine array. The result depicted that only three chemokines Eotaxin-1/CCL11, SDF-1α /CXCL12, and G-CSF were present at different levels between CD and ITB patients. Eotaxin-1/CCL11 was noted lower in TB than CD. Previously this molecule has been evaluated as biomarker in leprosy^29^ and Asthma^30^. To our surprise, studies suggest that the presence of Eotaxin-1/CCL11 triggers Th2 immune response as CCR3 receptor expresses on Th2 lymphocytes^31^. While Das et al. has documented that granuloma and/or non-granuloma CD and ITB both show more Th1 response^28^. The level of SDF-1α/CXCL12 was found exactly opposite to that of Eotaxin-1/CCL11. It is a very efficient chemo-attractant for lymphocytes and monocytes functioning through the CXCR4 receptor. This has been reported as biomarker for dyslipidemia^32^. Hence, ROC analysis revealed sensitivity and specificity of SDF-1α/CXCL12 as a single marker was better than Eotaxin-1/CCL11 or G-CSF alone while upon addition of either Eotaxin-1/CCL11 or G-CSF, diagnostic sensitivity reached to 77% and 92% with specificity of 80% and 60% respectively. The combination of Eotaxin-1/CCL11 + SDF-1α/CXCL12 + G-CSF had slightly better sensitivity and specificity of 85% and 72% respectively. Next, different combinations of mir-375-3p, and Eotaxin-1/CCL11, SDF1α/CXCL12, and G-CSF were tested considering the diagnostic potential of multiple markers could be better than a single one as CD and ITB both are heterogeneous complex diseases. Here, the combination of (miR-375-3p + Eotaxin-1 /CCL11 + SDF1α /CXCL12) and (miR-375-3p + Eotaxin-1 /CCL11 + SDF1α /CXCL12 and G-CSF) showed 100% specificity, 56% sensitivity and PPV and NPV of 100% and 69% with 78% accuracy while combination of miR-375-3p+G-CSF+SDF1α/CXCL12 showed 83% specificity, 61% sensitivity, 79% PPV, 68% NPV and 72% accuracy.

In summary, using two distinct molecular screening methods, four markers are identified in this study. Considering the complexity and heterogeneous nature of the diseases, a combination of four markers may be better to diagnose these two indistinguishable diseases. The data presented here is our preliminary insights with low number of samples, but the high specificity suggests validation in a new larger cohort of samples may improve the potential of this combination of plasma markers in differentiating ITB and CD.

## Materials and methods

### Ethical statement

Human Ethics Committee of the Institute of Post-Graduate Medical Education and Research (IPGME&R), Kolkata has approved the study, and prior written informed consent was obtained from each individual. All the experiments were performed in accordance with relevant guidelines and regulations.

### Inclusion of patients

Patients visiting the outdoor of Gastroenterology Department at School of Digestive and Liver Diseases, IPGME&R and R.G. Kar Medical College and Hospital, Kolkata during the period of January 2017-January 2020 for the evaluation of their gastrointestinal complications suspecting of IBD or ITB were included in the study. After verification of the clinical data fifty-two treatment naïve patients within the age group of 18 to 60 years were further evaluated for this study. Following the guidelines of European Crohn’s and Colitis after verification of the clinical, endoscopic, and histological features, twenty-five patients were included in the CD group (n = 25), while patients with necrotizing granulomas in histology, positive for PCR with MTB primers (MPB 64 and IS6100) and follow up data of increasing body weight after anti-tubercular therapy (ATT) were included in Intestinal Tuberculosis (ITB) group (n = 27). CD patients were negative in confirmatory TB-PCR with MTB primers and did not respond to ATT. In addition, 15 UC patients and three hemorrhoid individuals diagnosed from clinical, endoscopic, and histological observations were included as a control group.

### Collection of tissue and blood

Biopsy tissue of the Colon, Ileal, and Ileocaecal region from the patients was collected during colonoscopic evaluation of patient. Tissues were collected in RNA later (Life Technologies, Carlsbad, CA, USA) and in 10% formalin immediately. Samples collected in RNA later were kept at 4 °C for 24 h to allow the RNA later to penetrate and then tubes were preserved at − 80 °C for future use. Tissues collected in formalin were kept at room temperature and used for paraffin block preparation.

Peripheral blood (8 ml) was collected from each patient in the presence and absence of anti-coagulant to separate plasma and serum respectively by centrifugation at 5000 rpm for 20 min at room temperature. Plasma was further purified by centrifugation at 13,000 rpm for 5 min with the supernatant of the previous step. Both serum and plasma were then preserved at − 20 °C freezer in small aliquots for future use.

The paraffin-embedded tissue was sectioned of about 3 µm in thickness and stained with Hematoxylin and Eosin (H&E). The activity of the diseases was determined by the presence of neutrophil infiltration, crypt abscess, crypt architectural distortion and scored by two experienced pathologists independently.

### Genomic DNA isolation

Genomic DNA was isolated from 0.5 mg of tissue after overnight digestion with proteinase K followed by treatment with phenol–chloroform (1:1). Genomic DNA was precipitated with 2.5 volume ethanol and dissolved in 50 μl of DNase/RNase free water.

### PCR for detection of *Mycobacterium tuberculosis*

A PCR test was conducted to confirm the presence of TB by amplifying MPB64 and IS6110 sequences using the genomic DNA. Primer sequences are presented in Supplementary Table 3.

### RNA isolation from tissue and plasma

Total RNA was isolated from 0.5 mg of colon tissue using Trizol (Thermo Scientific) following the manufacturer’s protocol. In brief, the colon tissue was homogenized in 500 μl of Trizol, 1/5th volume of chloroform was added, and centrifuged. Isopropanol was added half of the volume of the Trizol for the precipitation of RNA, and washed with 70% ethanol, air dried and dissolved in RNase free water. RNA integrity (RIN) was assessed using Agilent 2100 Bio-analyzer.

Plasma RNA was isolated from 200 µl of plasma using miRNeasy serum/plasma kit following manufacturer’s protocol.

### Small RNA sequencing and differential expression analysis

Differential expression analysis of miRNAs in CD and ITB colonic tissues was performed by next-generation sequencing (NGS) analysis using Illumina HiSeq2500 platform in Medgenome Labs Private Limited, Bangalore, India. In brief, the libraries were prepared using total RNA isolated from colonic tissues of CD (n = 3) and ITB (n = 3) patients and ligated to 3′ and 5′ adaptor followed by reverse transcribed and amplified using Trueseq small RNA Sample Prep Kit. The sample was then subjected to small RNA sequencing with a read length of 50 nucleotides from one end.

To get the clean data, the low-quality reads and the adapter sequences were removed. The extracted data from the NGS is presented in Supplementary Table 2. The NGS data has been submitted to GEO database. The filtered sequences were aligned to the hg19/GRCh37 human genome reference sequence using mapper.pl of the miRDeep2 module.The length of reads, chromosome number, genomic position of the mapped sequence, chromosome strand, and mismatches were determined using the mapper.pl. The .arf file was generated for expression profiling in quantifier.pl script and microRNA was detected using the miRDeep2.pl script. Differentially expressed miRNAs were identified using DESeq2 packages and data with adjusted p-value < 0.05 was considered as significantly deregulated miRNAs in the sample.

### Quantitative RT-PCR analysis for validation

About 500 ng of total RNA was used for the synthesis of cDNA using miScript PCR starter kit (Qiagen) and real-time PCR was performed using SYBR green PCR master mix (Thermo Scientific) in ABI Quant Studio 7 Flex Real-Time PCR machine. The log value was [log_10_ (2^−ΔCt^ × 10^5^)] plotted. RNU6B and *C.elegans* were used as an internal control for quantification of miRNAs in tissue and blood respectively. Sequences of the primers are presented in Supplementary Table 3.

### BioplexPro^TM^Human cytokine screening panel assay

A BioPlexPro^TM^Human cytokine screening panel (Bio-Rad) was used to detect the levels of 48 types of serum Cytokines, and Chemokines. In brief, premixed beads coated with the 48 types of target capture antibodies were transferred into a 96-well filtration plate supplied with the assay kit. After two times washing with the Bio-Plex wash buffer, premixed standards and 50 µL of samples were added to each well, and incubated for an hour on a shaker (850 ± 50 rpm) at room temperature. Then it was washed with 100 µL Bio-Plex wash buffer, mixed with biotinylated detection antibodies (50 µL of 2 µg/mL) and incubated for 10 min on a shaker at room temperature. After washing, the beads were re-suspended in 125 µL of Bio-Plex assay buffer and read on a Bio-Plex array reader. The data were analyzed using BioPlex® 200 Multiplexing Platform.

### Statistical analysis

ROC and Statistical analysis were performed using R software packages, and GraphPad Prism 7 (La Jolla, CA, USA) respectively. For comparison between the two groups, Mann–Whitney t-test and κ^2^ analysis was performed as it was required. qRT-PCR data are presented as Mean ± SD. A value of p ≤ 0.05 was considered statistically significant.

## Supplementary Information

Supplementary Information.

## Data Availability

The NGS data of total RNA of three normal, three CD and three ITB tissue samples are available publicly in the GEO database with the identification number of GSE176255. Other data will be made available from the corresponding author upon reasonable request.

## Abbreviations

ANCA: Anti-neutrophil cytoplasmic antibodies
ASCA: Anti-*Saccharomyces cerevisiae* antibodies
ATT: Anti-tubercular treatment
AUC: Area under curves
CD: Crohn’s disease
CI: Confidence interval
CRP: C-Reactive protein
G-CSF: Granulocyte colony stimulating factor
HIV: Human immunodeficiency virus
IBD: Inflammatory bowel disease
ITB: Intestinal tuberculosis
LOXL-2: Lysyl oxidase homolog 2
miRNA: Micro RNA
MTB: *Mycobacterium tuberculosis*
NGS: Next generation sequencing
NPV: Negative predictive value
PCR: Polymerase chain reaction
PPV: Positive predictive value
pTB: Pulmonary tuberculosis
qRT-PCR: Quantitative real-time PCR
RIN: RNA integrity
ROC: Receiver operating curve analysis
SDF-1α: Stromal cell derived factor-1α
