# Supplementary material for: A combination of circulating microRNA-375-3p and chemokines CCL11, CXCL12, and G-CSF differentiate Crohn’s disease and intestinal tuberculosis
Source: Sci Rep. 2021 Dec 2;11:23303. doi: 10.1038/s41598-021-02383-z (PMC8639680; doi:10.1038/s41598-021-02383-z)
Supplement: Supplementary file 1 — Supplementary Information. [file 41598_2021_2383_MOESM1_ESM.pdf]

A combination of circulating microRNA-375-3p and chemokines CCL11, CXCL12, and G-CSF differentiate Crohn's Disease and Intestinal Tuberculosis

Susree Roy[1#], Suchandrima Ghosh[1#], Mallica Banerjee[1], Sayantan Laha[2], Dipanjan Bhattacharjee[3], Rajib Sarkar[4], Sujay Ray[5], Arka Banerjee[4], Ranajoy Ghosh[6], Aniket Halder[6], Alakendu Ghosh[3], Raghunath Chatterjee[2], Simanti Datta[1], Gopal Krishna Dhali[4], and Soma Banerjee[1*]

[#] Both the authors have contributed equally.

[1]Center for Liver Research, Institute of Post Graduate Medical Education and Research, Kolkata

[2]Department of Human Genetics, Indian Statistical Institute, Kolkata

[3]Department of Rheumatology, Institute of Post Graduate Medical Education and Research, Kolkata

[4]Department of Gastroenterology, Institute of Post Graduate Medical Education and Research, Kolkata

[5]R.G. Kar Medical College and Hospital, Kolkata

[6]Department of Gastro-Pathology, Institute of Post Graduate Medical Education and Research, Kolkata

*Corresponding Author : Soma Banerjee
Professor
Center for Liver Research
School of Digestive and Liver Diseases
Institute of Post Graduate Medical Education and Research
244, Acharya Jagadish Chandra Bose Road
Kolkata-700020
West Bengal, India.
Mobile No: 9748000673
E.mail: somabanerjee70@gmail.com

Running title: Biomarker to distinguish Intestinal Tuberculosis and Crohn's Disease

Supplementary Table 1: Clinical, demographical parameters of UC and Hemorrhoids patients included in the study.

Variable	Normal (n=3)	UC (n=15)

Baseline characteristics		
**Age(yr) (Mean±sd)**	42±18.7	42.7±10.8
**Sex (F/M)**	2/1	7/8
**Fever (Y/N)**	2/1	5/10
**Pain Abdomen (Y/N)**	1/2	13/2
**Weight Loss (Y/N)**	0/3	6/9
**Clinical characteristics**		
**Hemoglobin (gm/dl) (Mean±sd)**	11.6±2.7	10.1±1.5
**ESR (mm) (Median, Range)**	40 (35-42)	35 (12-150)
**CRP(mg/dl) (Median, Range)**	4.3 (2-36)	6 (1.5-36)

Supplementary Table 2: Extracted data from Next Generation Sequence analysis of CD, ITB and hemorrhoids (Normal) samples.

Sample	Raw Reads	Mapping to hg19 (%)	Mapping to mature miRNA (%)
CD1	33544611	98.17%	74.31%
CD2	26315327	98.50%	66.94%
CD3	23860962	98.61%	72.58%
ITB1	23922043	97.80%	71.87%
ITB2	32273807	98.45%	62.92%
ITB3	27313209	98.64%	46.05%
N1	27214031	92.13%	49.76%
N2	25868238	98.11%	59.31%
N3	27058058	97.14%	75.57%

Supplementary Table 3: Sequences of the Primers used in the study.

Name of the gene	**5'- 3' direction**

miR-31-5p	ACACTCCAGCTGGGAGGCAAGATGCTGGCA
miR-215-5p	ACACTCCAGCTGGGATGACCTATGAATTGA
miR-375-3p	ACACTCCAGCTGGGTTTGTTCGTTCGGCTC
miR-29a-3p	ACACTCCAGCTGGGTAGCACCATCTGAAAT
miR-615-3p	ACACTCCAGCTGGGTCCGAGCCTGGGTCTC
MTB IS6110F	CCT GCG AGC GTA GGC GTC GG
MTB IS6110R	CTCGTCCAGCGCCGCTTCGG
MPB64 F	TCCGCTGCCAGTCGTCTTCC
MPB64R	GTCCTCGCGAGTCTAGGCCA

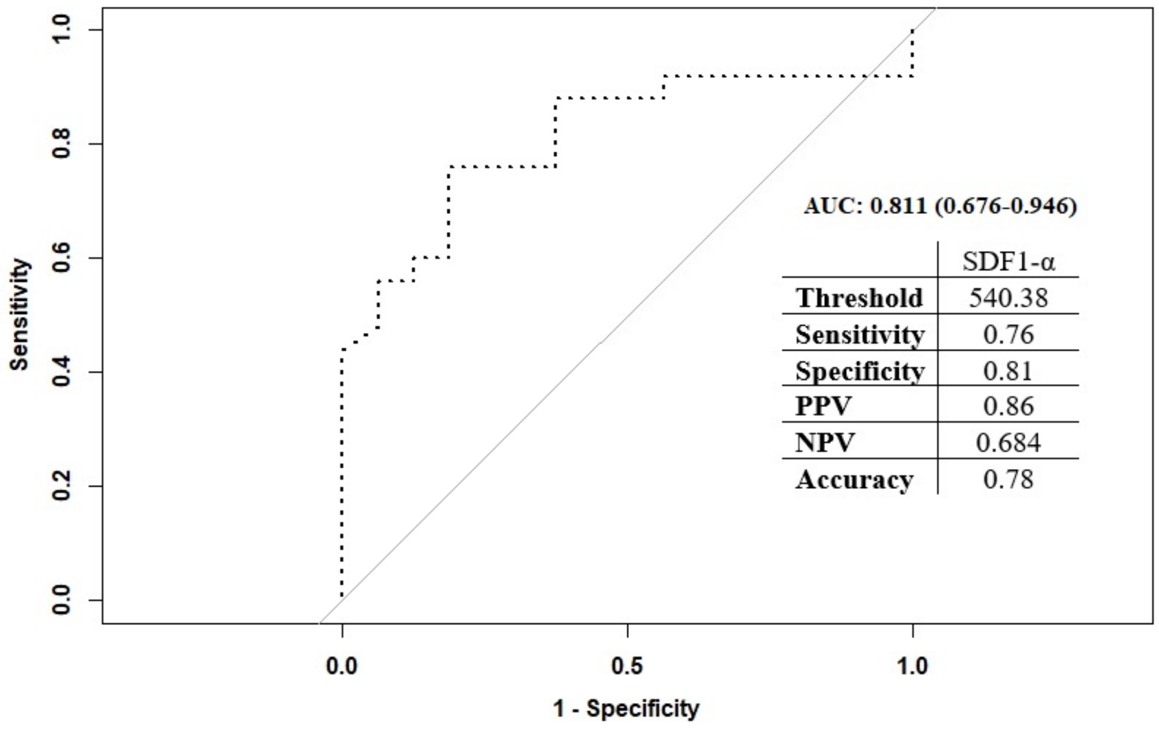

Supplementary Figure 1

Supplementary Figure 1: ROC analysis for SDF-1α/CXCL12 in differentiating CD and UC.
